# Supplementary material for: TRIM37 recognizes a bipartite degron to ubiquitinate centrosome substrates
Source: bioRxiv. 2025 Dec 21:2025.12.19.695442. Preprint. [Version 1] doi: 10.64898/2025.12.19.695442 (PMC12724674; doi:10.64898/2025.12.19.695442)

bioRxiv preprint doi: https://doi.org/10.64898/2025.12.19.695442; this version posted December 21, 2025. The copyright holder for this preprint (which was not certified by peer review) is the author/funder, who has granted bioRxiv a license to display the preprint in perpetuity. It is made available under aCC-BY 4.0 International license.

TABLE S1. Baculovirus constructs protein expression.

protein	sequence	plasmid name	parent plasmid	N-term tag	C-term tag
Cep192	Full-length	JWV99	pOCC29	MBP-PreScission -**eGFP**	PreScission-6xHis
Cep192 C-term	a.a. 2070-2537	JWV106	pOCC27	MBP-PreScission	**eGFP**-PreScission-6xHis
TRIM37 C18R	Full-length	JWV130	pOCC27	MBP-PreScission	**eGFP**-PreScission-6xHis

TABLE S2. Bacterial constructs for protein expression.

protein	plasmid name	parent plasmid	N-term tag	C-term tag
GFP	JWB66	pET	6xHis-GFP-TEV	
EB1	JWB3	pTEMM1	6xHis-TEV	**eGFP**
Cep192 C-term	JWB194	JWB66	6xHis-**eGFP**-TEV	
Cep192 C-term ΔASH6	JWB210	JWB66	6xHis-**eGFP**-TEV	
Cep192 C-term ΔASH7	JWB206	JWB66	6xHis-**eGFP**-TEV	
Cep192 C-term ΔASH8	JWB208	JWB66	6xHis-**eGFP**-TEV	
IDR + ASH8	JWB223	JWB66	6xHis-**eGFP**-TEV	
ASH8	JWB225	JWB66	6xHis-**eGFP**-TEV	

TABLE S3. Constructs for protein expression using TREx Flp-In™ system in DLD-1 human colon cancer cells.

Protein expressed	mutation	Cell line name	parent plasmid
GFP::Cep192	WT	JWM25	JWB179
GFP::Cep192	7KtoR	JWM24	JWB180
GFP::Cep192	no ASH8 (1-2437aa)	JWM28	JWB224

bioRxiv preprint doi: https://doi.org/10.64898/2025.12.19.695442; this version posted December 21, 2025. The copyright holder for this preprint (which was not certified by peer review) is the author/funder, who has granted bioRxiv a license to display the preprint in perpetuity. It is made available under aCC-BY 4.0 International license.

GFP::EB1	WT	JMM 29	JWB237
GFP::EB1::ASH8	IDR_ASH8	JMM 30	JWB230

Extended Data Set 1. Mass spectrometry data for detection of ubiquitinated sites on Cep192.

Extended Data Set 2. Cross-linking mass spectrometry data.

bioRxiv preprint doi: https://doi.org/10.64898/2025.12.19.695442; this version posted December 21, 2025. The copyright holder for this preprint (which was not certified by peer review) is the author/funder, who has granted bioRxiv a license to display the preprint in perpetuity. It is made available under aCC-BY 4.0 International license.

Figure S1. Identification of ubiquitinated residues and the TRIM37-binding site on Cep192.

A) Domain architecture of TRIM37.

B) SDS-PAGE gel showing purified full-length Cep192 and fragments.

C) List of ubiquitinated lysines identified by MS and reported in PhosphoSitePlus (https://www.phosphosite.org/). Lysines highlighted in yellow were found to be ubiquitinated in our samples and in PhosphoSitePlus.

bioRxiv preprint doi: https://doi.org/10.64898/2025.12.19.695442; this version posted December 21, 2025. The copyright holder for this preprint (which was not certified by peer review) is the author/funder, who has granted bioRxiv a license to display the preprint in perpetuity. It is made available under aCC-BY 4.0 International license.

D) qPCR analysis of construct expression across three biological replicates. Bars show mean with 95% CI; P values from one-way ANOVA followed Dunnett's multiple comparison.

E) Western blot showing binding efficiency of Cep192 constructs to TRIM37. Constructs were expressed inside DLD-1 cells and then pulled down using beads coated with TRIM37.

Figure S2. All Cep192 constructs localize to centrioles and support bipolar spindle formation.

A) Immunofluorescence of cells after 96 hr of tetracycline-induced expression of Cep192 constructs. GFP constructs (green), Centrin (representing centrioles; magenta) and DNA (blue). Black scale bar: 5 μm; white scale bar: 1 μm.

B) Representative images showing localization of GFP-Cep192 constructs in mitotic cells (outlined in white). Red arrows label chromosomes aligned at the metaphase plate. Scale bar: 5 μm.

Figure S3. Biochemical characterization of the IDR+ASH8–TRIM37 interaction.

A) Western blot against TRIM37 showing its abundance after siTRIM37 treatment.

B) Schematic of pull-down assay from Fig. 5B.

C) Western blot of TRIM37 found in the supernatant of experiments from Fig. 5B.

D) Cross-links from Fig. 5D mapped onto AlphaFold3 predicted structures of the ASH8 domain and a TRIM37 dimer (a.a. 1-458). Shown are cross-links that satisfy the maximum distance constraint for DSSO (35 Å; between alpha carbons).

E) DSSO crosslinking map of TRIM37 and IDR+ASH8 with calculated FDR <5% (medium confidence). ASH8 is colored orange; intrinsically disordered regions are colored white. Cross-links: purple, intramolecular; green, intermolecular; red, intermolecular links between the same amino acid. n = 3 biological replicates.

F) Overlay of ASH8 (AlphaFold3 model) with ASH7 (PDB: 6FVI) and ASH6 (AlphaFold3 model).

bioRxiv preprint doi: https://doi.org/10.64898/2025.12.19.695442; this version posted December 21, 2025. The copyright holder for this preprint (which was not certified by peer review) is the author/funder, who has granted bioRxiv a license to display the preprint in perpetuity. It is made available under aCC-BY 4.0 International license.

G) Estimated surface charge of structures in panel F (blue, positive; red, negative).

bioRxiv preprint doi: https://doi.org/10.64898/2025.12.19.695442; this version posted December 21, 2025. The copyright holder for this preprint (which was not certified by peer review) is the author/funder, who has granted bioRxiv a license to display the preprint in perpetuity. It is made available under aCC-BY 4.0 International license.

Figure S1

A

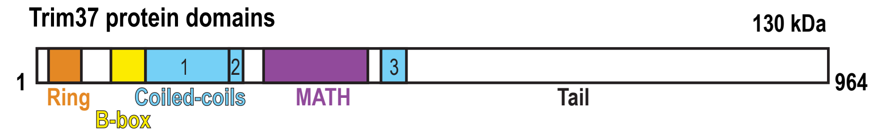

B

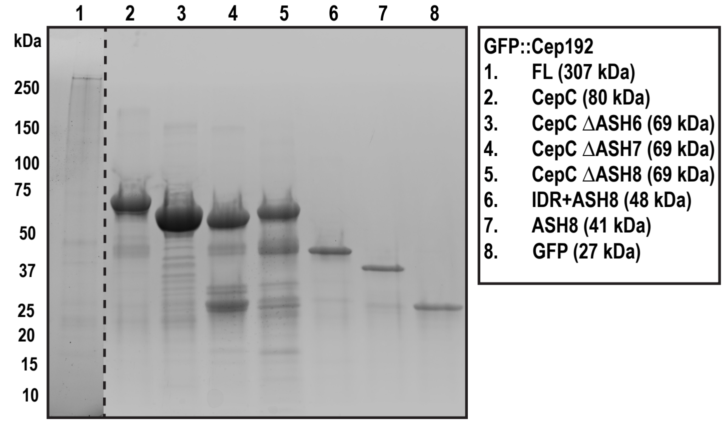

C

Identification				Abundance		
no	identified Ub site	by MS	by phosphosite	Average positive sample	Average negative sample	difference
1	K1602	yes	yes	6.62E+04	6.72E+04	-9.15E+02
2	K1661	yes	yes	1.02E+05	9.04E+04	1.18E+04
3	K1752	yes	yes	2.35E+04	1.48E+04	8.65E+03
4	K1870	yes (2 different peptides)	no	5.55E+04	4.76E+04	7.99E+03
5	K1890/91	yes	yes	1.45E+05	9.68E+04	4.81E+04
6	K1926	yes	yes	3.01E+05	2.75E+05	2.65E+04
7	K1975	yes	yes	2.82E+05	1.88E+05	9.42E+04
8	K2078	yes	yes	1.14E+05	9.91E+04	1.50E+04

D

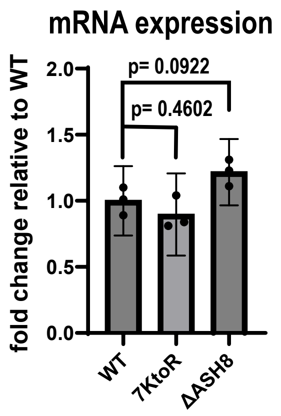

E

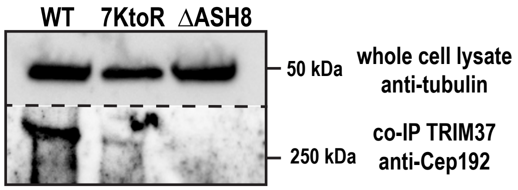

bioRxiv preprint doi: https://doi.org/10.64898/2025.12.19.695442; this version posted December 21, 2025. The copyright holder for this preprint (which was not certified by peer review) is the author/funder, who has granted bioRxiv a license to display the preprint in perpetuity. It is made available under aCC-BY 4.0 International license.

Figure S2

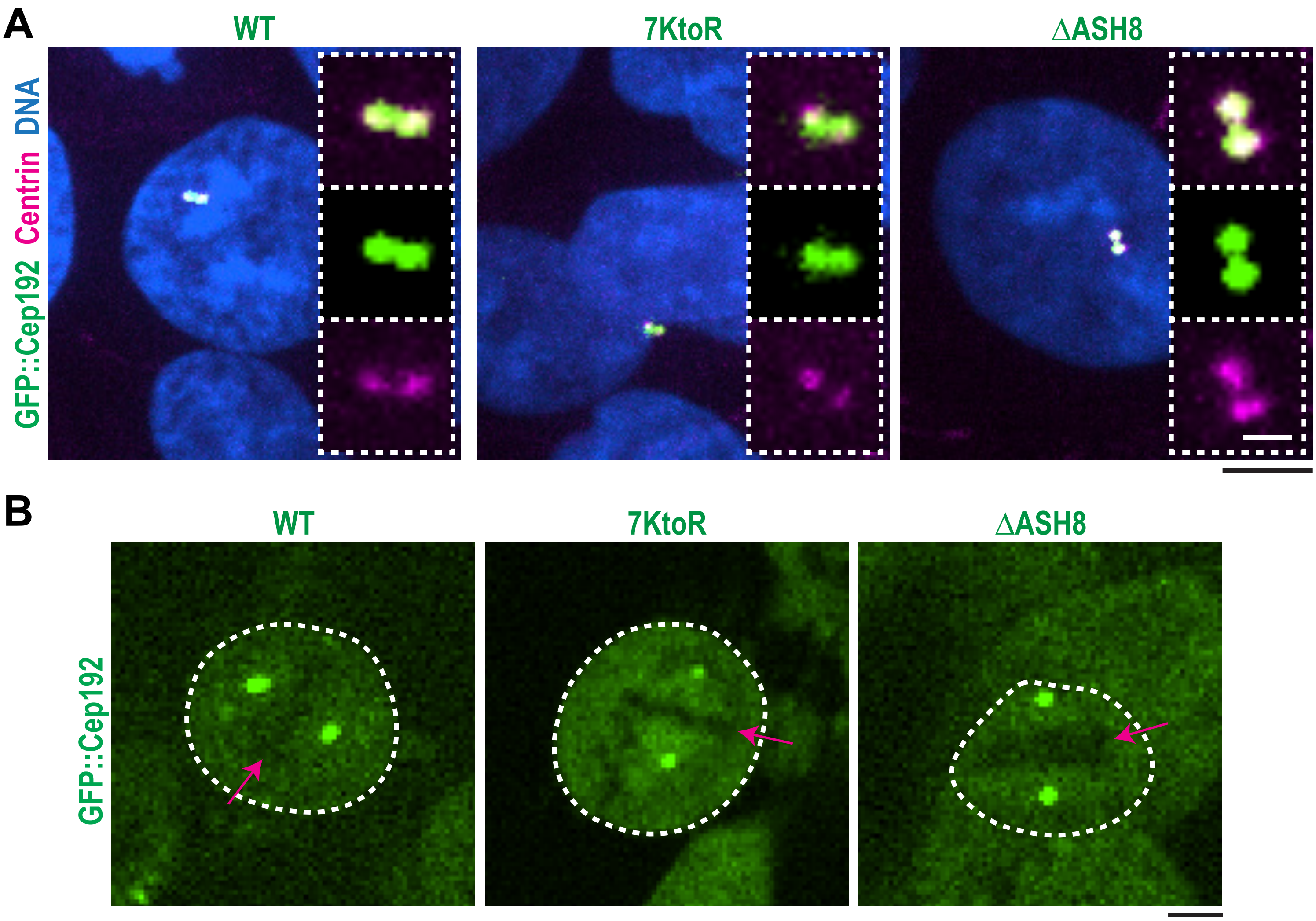

bioRxiv preprint doi: https://doi.org/10.64898/2025.12.19.695442; this version posted December 21, 2025. The copyright holder for this preprint (which was not certified by peer review) is the author/funder, who has granted bioRxiv a license to display the preprint in perpetuity. It is made available under aCC-BY 4.0 International license.

Figure S3

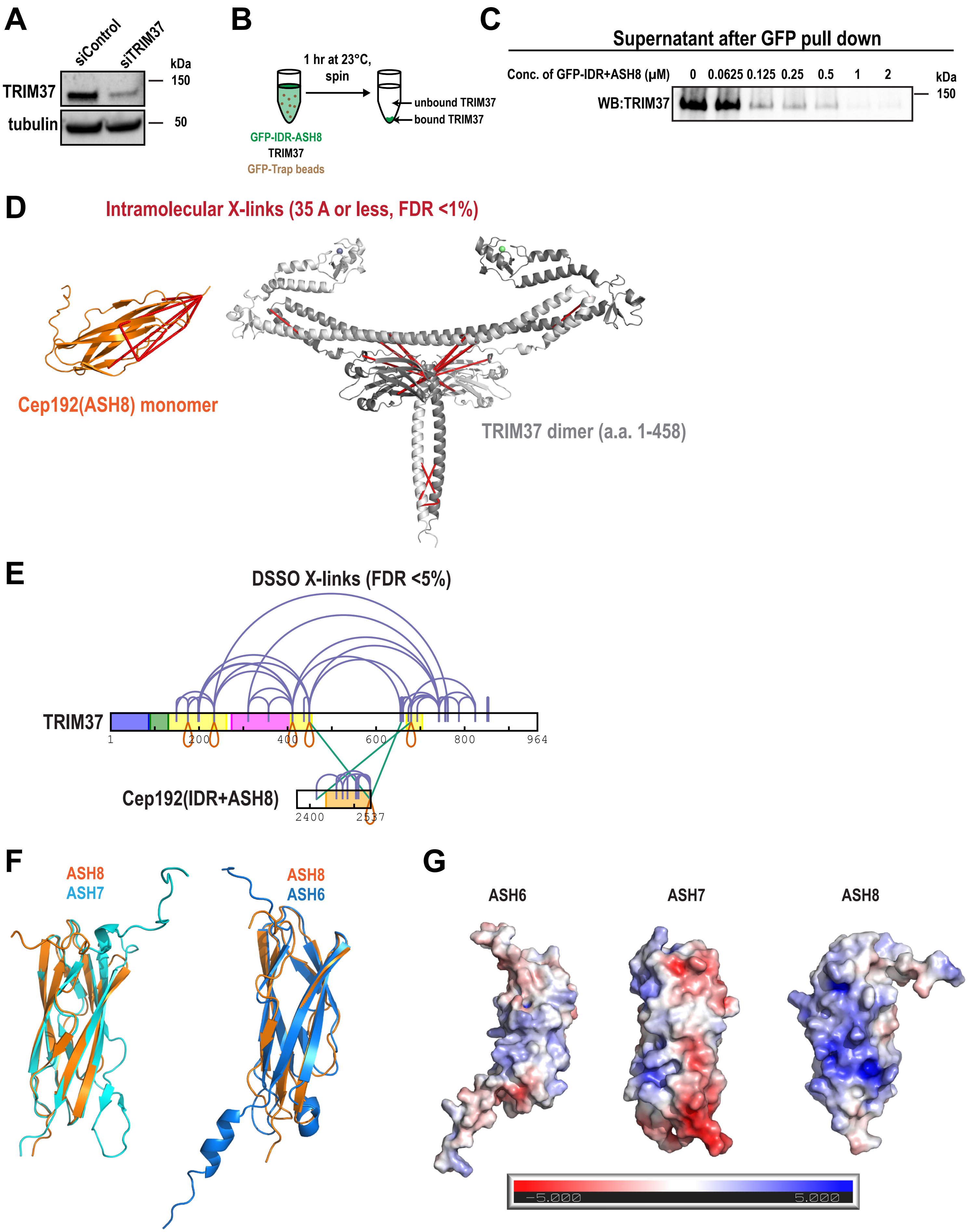

Supplement: 1 [file NIHPP2025.12.19.695442V1-supplement-1.pdf]
